# Preliminary Design and Experimental Investigation of a Novel Pneumatic Conveying Method to Disperse Natural Fibers in Thermoset Polymers

**DOI:** 10.3390/ma9070548

**Published:** 2016-07-07

**Authors:** Mahi Fahimian, Mark Kortschot, Mohini Sain

**Affiliations:** 1 Advanced Materials Group, Department of Chemical Engineering and Applied Chemistry, University of Toronto, 200 College St., Toronto, ON M5S 3E5, Canada; mahi.fahimian@utoronto.ca; 2 Center for Biocomposites and Biomaterials Processing, Faculty of Forestry, University of Toronto, 33 Willcocks Street, Toronto, ON M5S 3B3, Canada; m.sain@utoronto.ca

**Keywords:** natural fibers, composites, pneumatic conveyors

## Abstract

Natural fibers can be attractive reinforcing materials in thermosetting polymers due to their low density and high specific mechanical properties. Although the research effort in this area has grown substantially over the last 20 years, manufacturing technologies to make use of short natural fibers in high volume fraction composites; are still limited. Natural fibers, after retting and preprocessing, are discontinuous and easily form entangled bundles. Dispersion and mixing these short fibers with resin to manufacture high quality, high volume fraction composites presents a significant challenge. In this paper, a novel pneumatic design for dispersion of natural fibers in their original discontinuous form is described. In this design, compressed air is used to create vacuum to feed and convey fibres while breaking down fibre clumps and dispersing them in an aerosolized resin stream. Model composite materials, made using proof-of-concept prototype equipment, were imaged with both optical and X-ray tomography to evaluate fibre and resin dispersion. The images indicated that the system was capable of providing an intimate mixture of resin and detangled fibres for two different resin viscosities. The new pneumatic process could serve as the basis of a system to produce well-dispersed high-volume fraction composites containing discontinuous natural fibres drawn directly from a loosely packed source.

## 1. Introduction

Natural fiber reinforced composites have been an increasing focus of the materials research community over the past 20 years [1]. In addition to environmental benefits, they offer potential for both weight and cost savings when compared to conventional fiberglass composites [2]. However, natural fibre composites can present some unique processing challenges. Injection molded and extruded short fiber composites have been commercialized [3], but manufacturing technologies for high volume fraction composites with longer fibers are still being developed [4,5].

Part of the challenge of commercializing natural fibres is the inherent variability of fiber properties due to variations in the environment and harvest timing [6]. There are efforts to address this issue by controlling the harvesting plan for industrial grade natural fibers [7] as well as sorting natural fibers and providing a comprehensive data base for fiber properties [8]. 

Another challenge impeding the more widespread use of natural fibers in high production composite applications is the difficulty in handling inhomogeneous fibres in automated processing equipment. Fibreglass composites are widely used in both construction and transportation industries. Twenty percent of overall fiber glass productions are for non-structural applications using open process such as spray lay-up [3,9]. In spray lay-up, continuous glass rovings are pulled through a chopper gun and cut by a rolling cartridge to a prescribed length. Chopped fibers are then dropped into the stream of sprayed resin, and the mixture is deposited on a mold. The fiber volume fraction is controlled by resin flow rate and adjusting the number of strands and the speed of the glass fibre roving. The fibre length is controlled by the numbers of cutters on the rolling cartridge. Discontinuous natural fibers such as hemp and flax have the potential to serve as the reinforcement in spray layup applications [1,10] but only if a method of feeding discontinuous fibres in a reliable way can be found.

Natural fibers might also be suitable for use in fiber reinforced polyurethane foams. Fibers can improve the mechanical strength [11,12] and potentially yield cost savings. However, mixing short fibers with the fast curing foams is a challenge that may limit the fraction of fibers in the final product. In laboratory studies, neither mechanical nor hand mixing were found to be efficient methods of dispersing fibre clumps in high viscosity resins [13]. 

**Figure 1:**
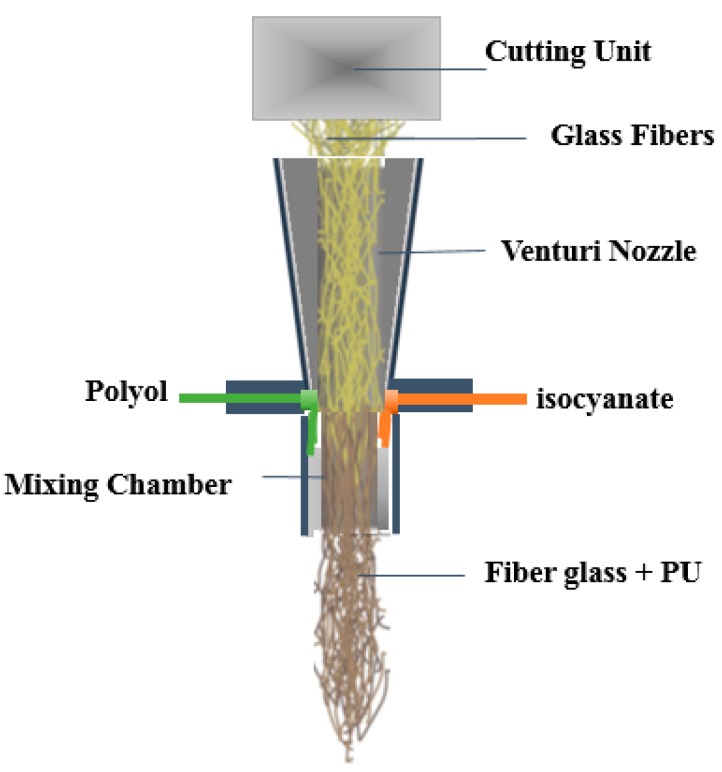
Long Fiber Injection (LFI) mixing head, Internal mixing of fiberglass and Polyurethane components in mixing chamber before injection [15].

Good dispersion has been achieved by a process called Long Fiber Injection (LFI), introduced in late nineties by Krauss Maffei [14]. In this method, a fiber roving is fed to a mixing head, and chopped as in a conventional fiberglass system, but instead of mixing with the sprayed resin externally, fibres are combined with polyurethane in a mixing chamber just before discharge to the mold (see Figure 1). The LFI method is useful for manufacturing large parts for the automobile industry because of a rapid cycle time, and its ability to make large parts from short fiber composites with volume fractions up to 40% and localized control over the volume fraction distribution [14]. 

Although both LFI and fiberglass spray lay-up use long fiber roving as the raw material, the fiber length in final product is cut to the range of 12–100 mm. Natural fibers extracted from wood or agricultural crops, after degradation and fiber separation processes, are usually discontinuous and quite short. Manufacturing continuous rovings (or yarns) from short fibers adds a manufacturing step and increases the cost of the overall process. Natural fibre yarns are made by twisting shorter fibers, and they are typically thicker compared to glass rovings and can have multiple defects along their length (some created by twisting process and some are created during the growth of the plant). These defects create weak spots [16] that can limit the pulling force applied to the roving system and therefore reduce the maximum feeding rate in a Spray lay-up and LFI process. 

Natural fibers are more ductile than glass fibers, and therefore the cutting mechanism in the chopper must be modified [15]. Finally, natural fibres that are too short or too stiff to be twisted into rovings might still be very suitable reinforcing agents, provided suitable manufacturing technology can be developed. 

In order to incorporate short natural fibers into a composite manufacturing process such as spray lay-up without first creating a roving, a feeding system that can both feed and meter the fibers reliably is needed. Hussain and Kortschot introduced a novel design for metering short form natural fibers with constant mass flow rates [17]. Their design consisted of a constant pressure roll nip in which the feeding speed was controlled to be inversely proportional to the nip gap. For example, if a big clump of fibers passed through the nip between the wheels, the nip speed was instantaneously decreased to ensure constant mass flow. However, in their design of the feed system, conveying fibers to the roll nip and mixing them with resin stream after the nip were not addressed. 

In this paper, a novel design for delivering a controlled amount of natural fibers to a stream of sprayed resin is introduced. In this design, the fibers are used in their short form and are conveyed and dispersed using a novel pneumatic conveying system. The pneumatic design uses compressed air to create vacuum for conveying the fibers. In addition, the force of compressed air breaks fiber clumps and detangles and disperses them. Lab scale equipment using these processes was set up and the viability of the process investigated by manufacturing model composite samples using Medium Density Fiberboard (MDF) fibers as reinforcing fibers. Vinyl ester and polyurethane were used as resins for manufacturing composite samples, which were used only to evaluate the ability of the system to detangle the MDF fibres and provide a good dispersion of fibres and resin. Optical microscopy and X-ray tomography images were used to evaluate dispersion and mixing. The model composites were not tested for mechanical properties or structural integrity in the present study.

## 2. Conceptual Design

The design of a natural fiber dispersion system is, at its core, a material conveying problem. The goal is to transfer a metered amount of short fibers continuously to a stream of resin in a way that guaranties good dispersion of fibers.

The design can be divided into three main components: feed, conveying and dispersion units. The feed unit must extract loose packed short fibres from boxes or bags and provide a continuous flow of fibers to the conveying system. Natural fibres with aspect ratios of more than 10 and rough surfaces typically form tangled agglomerates [18], so the conveying system needs to move the fibres and also break up the fibre agglomerates to deliver a controlled mass flow of well separated fibers to the dispersion unit. In the dispersion unit, individual fibres must be mixed with a stream of sprayed resin and the mixture is then sprayed onto or injected into a mold.

It is worth mentioning that breaking fiber agglomerates is an important component of the design since it is necessary in order to produce a composite with well-dispersed fibres of a known volume fraction. Proper fiber separation prior to the resin mixing operation affects the wetting of the fiber by resin and hence affects interfacial bond strength and mechanical properties of the manufactured composite part [19].

### 2.1. Feed

Like most bulk solids, short natural fibers can be transferred to a hopper. Capturing a continuous flow of fibers from a hopper containing tangled fibre agglomerates can be a challenge. Bridging and clogging the discharge port of the hopper is an issue in many bulk material handling operations, and a variety of techniques, including vibration and mechanical agitation to break the fiber clumps can be advantageous in providing a consistent feed [20,21]. In case of natural fiber dispensers, the size of discharge is limited by the amount of fibers needed to make up a certain volume fraction of composites. In this design, the application of a metering system developed previously by Hussain and Kortschot [17] has been evaluated. Using this metering unit introduces two challenges. One is feeding the fibers to the nip of the metering wheels. Because of entanglement of natural fibers, they bridge the entrance to the feeder roll nip unless the dimensions are large enough to ensure bridge collapse. Bridging characteristics of each bulk material depends on its inherent surface and geometrical characteristics. Therefore, it is essential to experimentally evaluate bridging characteristics of the fibers of interest to find the optimal discharge size for the feed.

The second challenge is stripping the fibers from the roll nip exit and transferring them to the conveying system. The simple roll nip metering system draws in fibre agglomerates and feeds them compressed but intact, albeit with roll speed adjusted to ensure constant mass flow. In order to create a suitable feed for conveying and spraying the fibres, individual fibres must be stripped from the exit of the roll nip as the agglomerate emerges. 

### 2.2. Convey

Receiving the feed of fibers from the hopper and delivering it to the dispersion unit is the responsibility of the conveying unit. A pneumatic conveying system has been introduced in this design. Since a pneumatic conveyor has few or even no moving parts, it can operate reliably without maintenance. In a pneumatic conveyor, the fibers are suspended in air with sufficient turbulence so as to maintain a stable suspension [22,23]. The required air velocity depends on the size, density, surface roughness and aspect ratio of the fibers. Natural fibers have lower density and rougher surfaces than glass fibres, and thus can be suspended at relatively low air velocity.

In this design an application of a novel in-line conveying unit manufactured by Exair Corp. (Cincinnati, OH, USA) has been adapted for use in conveying fibres. The unit can operate with as little as 5 Cubic Feet per Minute (CFM) of compressed air passing through a plenum chamber and then injected through series of directed nozzles. This action creates vacuum at the inlet section of the in-line vacuum, due to the venturi effect. The vacuum draws fibers into the chamber where they are mixed with turbulent, high velocity air and effectively transported while suspended in a dispersed state. The system has an additional advantage: clumps of natural fibers that emerge from the roll nip are effectively broken by the high turbulence near the injection ports and further downstream. 

**Figure 2:**
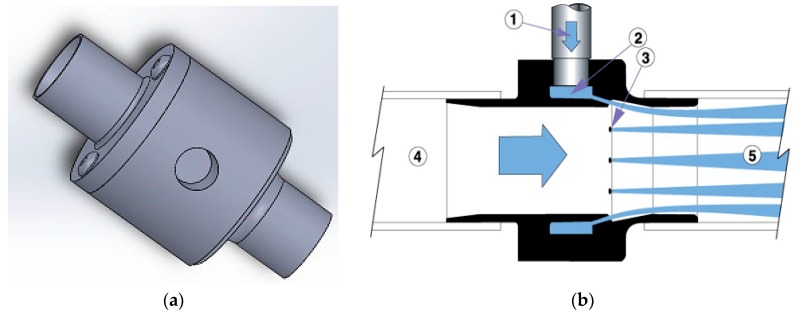
(**a**) 3D image of Exair in-line Vacuum; (**b**) Cross sectional view of in-line vacuum, Compressed air flows through the inlet (1) in to an annular plenum chamber (2) it is then injected through directed nozzles (3), the jets of air create a vacuum at the intake (4) which draws the fibers and accelerate them through the unit (5) at the same time the air pressure and flow breaks fiber clumps and disperse them in the air at (5). Image courtesy of Exair Corporation [24].

A schematic of the Line-Vac vacuum conveyor from Exair Corp. is shown in Figure 2. The compact size of Line-Vac (ranging from 7.62 to 50.8 mm in diameter) is suitable for incorporation in a small mobile fiber dispensing unit. The mass flow of material that is conveyed by this conveying unit depends on the density of material, supplied air pressure and the diameter of the in-line vacuum. Feeding a continuous, dispersed flow of fibers is essential, in order to minimize the variation in mass flow rate of fibers in the discharge of the in-line vacuum. 

The relation between air pressure and the discharge mass flow rate of fibers was measured experimentally in this design and the details are discussed in detailed design section.

### 2.3. Mix/Disperse

The system is intended to produce a well-mixed dispersion of wood fibres and liquid thermosetting resin. The stream of natural fibers and carrier air is mixed with stream of sprayed resin in the dispersion unit. In the present design, the fibers and resin are mixed in free air by directing two jets into a convergence zone very near the nozzles, very similar to some glass fibre chopper gun systems. One important difference between a fiberglass system and the present design is the presence of a large amount of excess air that is carrying the fibres. 

In principle, it might be possible to use an internal mixing method similar to that used in a Long Fibre Injection (LFI) process. However, in this paper, the model composite samples were manufactured using a very simple post-nozzle jet impingement to achieve an effective mixture of fibres and liquid resin. 

**Figure 3:**
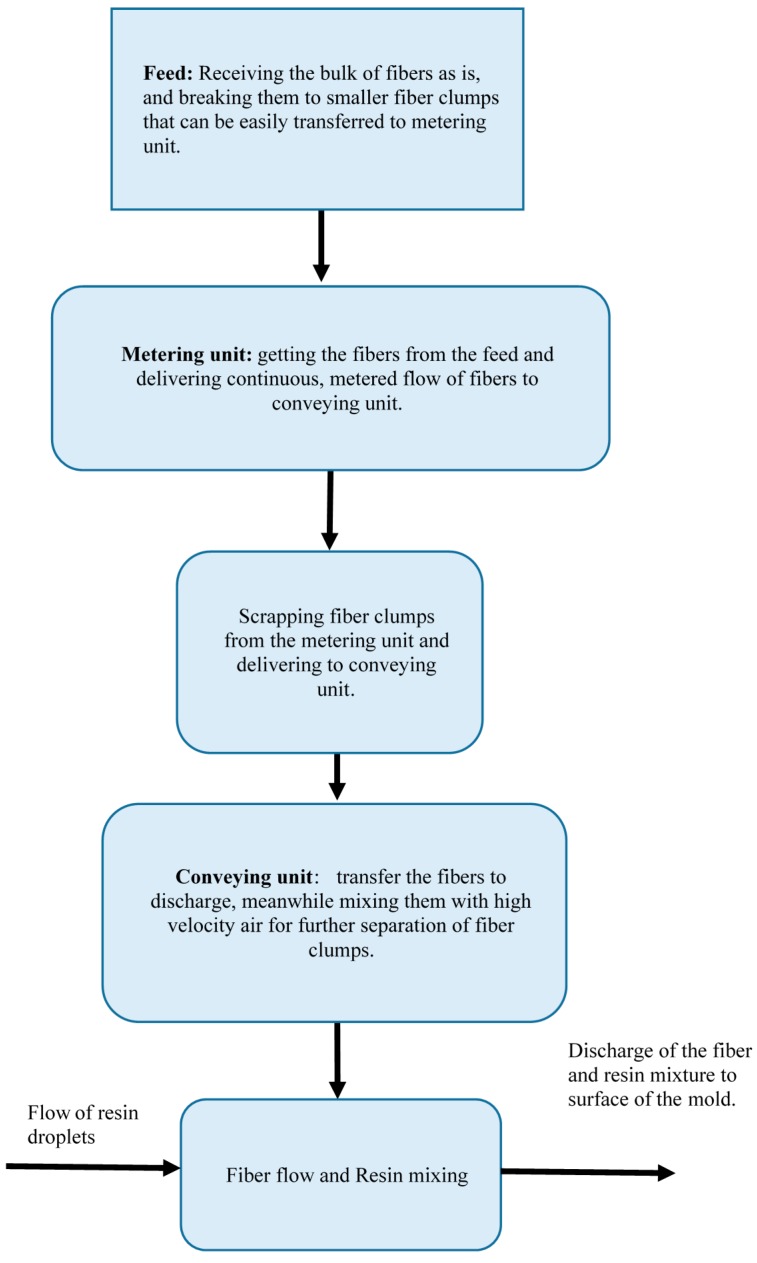
Flow chart of integrated unit design.

Figure 3 illustrates the design of the integrated unit.

## 3. Detailed Design and Supporting Experiments

The detailed design phase was supported by a series of experiments to determine the optimal configuration and process parameters. In this section, we describe both the details of a functioning prototype, as well as a few experiments required to make final design decisions. For all experiments, loose packed MDF fiber with aspect ratio of 10–20 and length ranging from 0.3 to 0.7 mm, was used as the natural fibre reinforcement in this study. The fibre was dumped into a hopper with a 5.44 kg (192 oz) capacity and a top OD of 0.2667 m (10.5′′). 

### 3.1. Feeding Unit

In order to design the diameter of the discharge port of the hopper, a set of experiments were done to study the bridging behavior of the MDF fibers used in this study. The MDF fibres were poured from a bag onto a plate with a circular holes ranging from 2.54 to 20.32 cm (1–8′′) in diameter. The purpose was to find out the minimum diameter that ensure no fibre bridging. For the MDF fibers used here, a 10.16 cm (4′′) diameter hole was the minimum hole that ensured fibers would pass through and not bridge. Unfortunately, the roll nip of the metering unit was smaller than this, meaning that it was difficult to obtain a consistent flow of fibres into the nip.

Mechanical agitation was also studied experimentally as a means of ensuring that fibres moved smoothly through the hopper outlet to the metering system. In principle, the diameter of the discharge port can be reduced by applying a mechanical agitation (stirring paddles) or pneumatic agitation (compressed air flow). In this study, a variety of agitation methods were tested, but only pneumatic agitation proved reliable. In the final prototype, pneumatic agitation with jets of pulsating compressed air (206.8 kPa source pressure) was used to agitate and break the fiber clumps in the hopper. Using this method, the outlet of the hopper was reduced to 5.08 cm (2′′) while continuous fiber flow was preserved. 

At the outlet to the hopper, a roll nip consisting of two open hand mixer beaters were turned at low speed to capture the fibres coming through the roll nip at something approaching consistent mass flow. In the prototype, the methods developed by Hussein and Kortschot [17] to deliver truly constant mass flow rate were not employed. 

### 3.2. Conveying Unit

**Figure 4:**
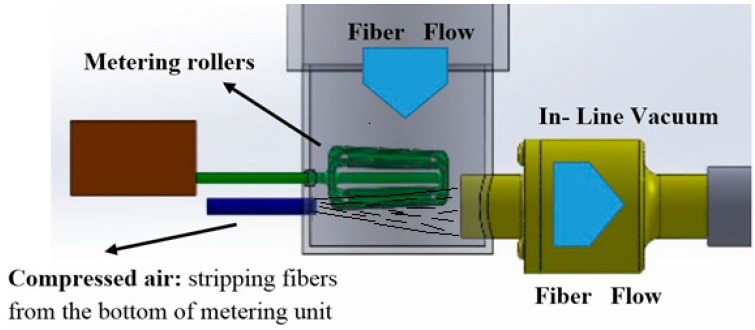
The schematic design of metering rollers, compressed air line and in-line vacuum.

Fibers were stripped from the bottom of the metering rollers by an intense jet of compressed air delivered at 551.6 kPa (80 psi). The jet was oriented parallel to the roller axis and positioned adjacent to the roll nip exit. The jet blew stripped individual fibres directly into the inlet of the in-line vacuum. Figure 4 shows the schematics of the design for location of metering rollers, in-line vacuum and compressed air line. A 2.54 cm inside diameter Line-Vac from Exair Corp. was used for capturing and conveying the fibres from the roll nip to the impingement mixer zone. Using a line pressure of 620.5 kPa (90 psi) and an airflow of 0.41 m^3^/min (14.5 CFM), the 2.54 cm diameter (1′′) Line Vac produced a vacuum suction of 11 kPa, and an exit airflow of approximately 0.85 m^3^/min (30 CFM), half of which came from the supply air and half of which was the entrained air stream loaded with fibres from the hopper.

**Figure 5:**
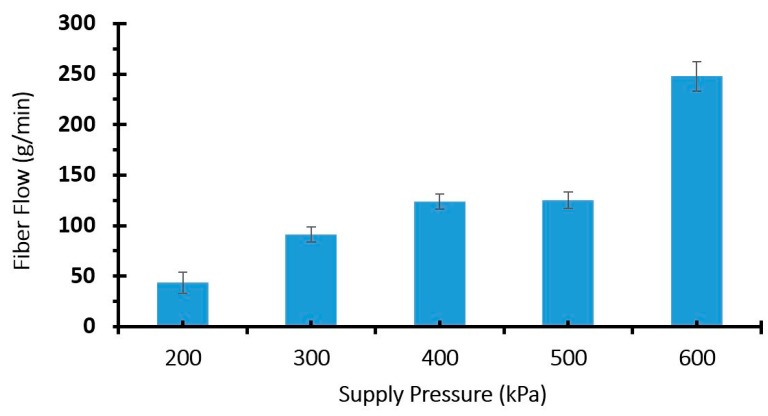
Correlation between supply air pressure and cumulative mass of fibers at the outlet of in-line vacuum.

An experiment was done to measure the relationship between mass flow rate of the MDF fibers in the outlet of the in-line vacuum and the inlet pressure used to drive the vacuum. A jet of compressed air (supply pressure of 206.8 kPa), agitated the fibers in the hopper near the inlet of the in-line vacuum to provide a consistent fibre density near the inlet. The mass of fibers drawn through the vacuum over 60 s was recorded. Five trials were done for each pressure. The relationship between the fibre flow rate and the inline vacuum supply air pressure is shown in Figure 5.

**Figure 6:**
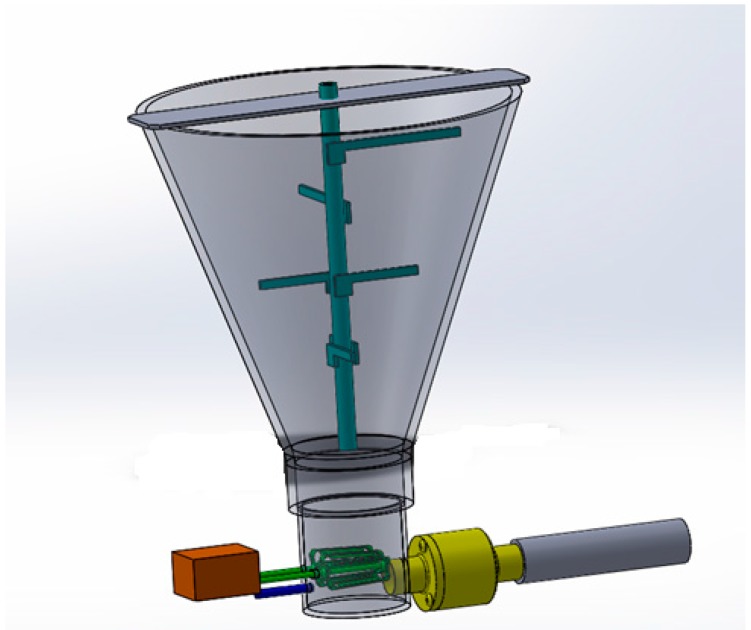
Schematic design of the fiber dispenser (feed plus conveying) unit.

As expected, an increase in the air pressure driving the vacuum increases the mass flow rate of air and entrained MDF fibers as well, (Figure 5). However, air pressure is not the only controlling factor effecting the outlet mass flow rate of the fibers, since the inlet air flow to in-line vacuum is still restricted by the hopper outlet flow. This implies that it might be possible to run the system without any mechanical metering device. However, in this study, a system of metering rollers at the outlet of hopper was also investigated. A schematic design of the fiber dispersion unit with metering rollers is shown in Figure 6. 

### 3.3. Mixing/Dispersion Unit 

**Figure 7:**
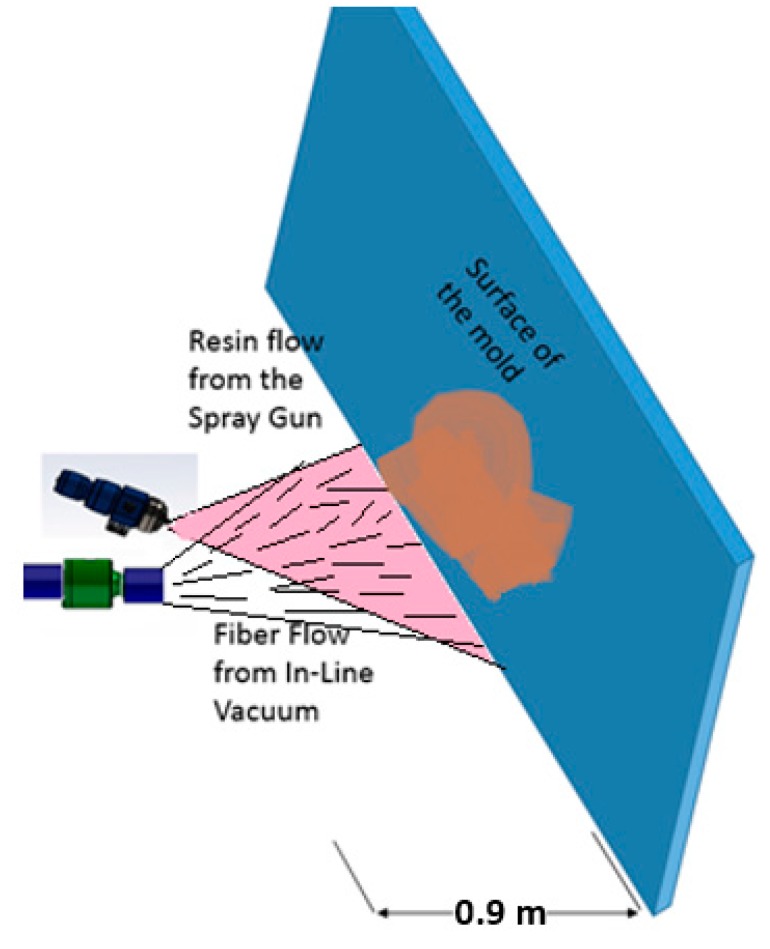
Schematic of the experimental set up of the fiber and resin external mixing.

The mixing of fibre and resin streams was accomplished by simply intersecting the two jets in front of a planar deposition surface, as illustrated in Figure 7. The mass fraction of fibres in the final composite material depends on the relative flow rates of the two streams and preliminary experiments allowed us to vary this ratio within limits. Other parameters that would influence the final composite quality include jet velocities, jet angles, jet spreading angle, impingement angle and so on. These parameters were not systematically varied in the current study. 

## 4. Proof-of-Concept Results and Discussion

A set of model composite samples were manufactured using the fibre dispenser depicted in Figure 6. For these trials, a 206.8 kPa (30 psi) pulsating compressed air jet was used to assist dispersion and flow in the hopper, the metering rollers were run at approximately 100 revolutions per minute (RPM), a jet of compressed air 206.8 kPa (30 psi) was used to strip fibres from clumps coming through the rollers, and the in-line vacuum was driven by line pressure of 413.7 kPa (60 psi), producing a total volumetric flow rate (driving + entrained air) of around 1.02 m^3^/min (36 CFM).

Mixing of fiber and resin was done externally in a jet impingement zone as shown schematically in Figure 7. The location of fiber and resin jets were varied until they produced a coherent and well mixed jet, but this aspect of the design was not formally studied. 

Two separate resins were used to test the spray system. In the first set of trials, Derakane M411-350 P vinyl ester (Ashland, Dublin, DE, USA) was used. The viscosity of the resin was 370 cps and its gel time was 20 min. An air operated spray gun, E.S. G100 (ES Manufacturing Inc., St. Petersburg, FL, USA) with 2 mm Nozzle tip was used to spray the vinyl ester resin. The spray gun consumed 0.14 m^3^/min (5 CFM) air at 551.6 kPa (80 psi) pressure. The jets were directed to a simple piece of flat plywood at a distance of about 40 cm. 

Vinyl ester-MDF mixtures were manufactured in 10%, 22% and 34% weight fractions of fibers by directing the air fibre stream from the dispersion unit into the vinyl ester aerosol jet as depicted in Figure 7. The weight fraction of fibres is simply the weight of fibres dispensed divided by the total weight measured after cure. In these experiments, no consolidation pressure was applied. The primary purpose for these experiments was to evaluate the dispersion of the fibers rather than to form a properly consolidated composite. 

**Figure 8:**
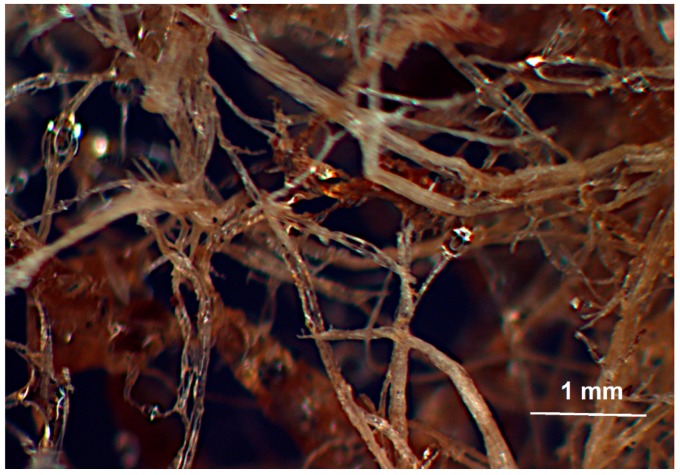
Optical Image of 10% Vinyester resin and Medium Density Fiberboard (MDF) fiber.

**Figure 9:**
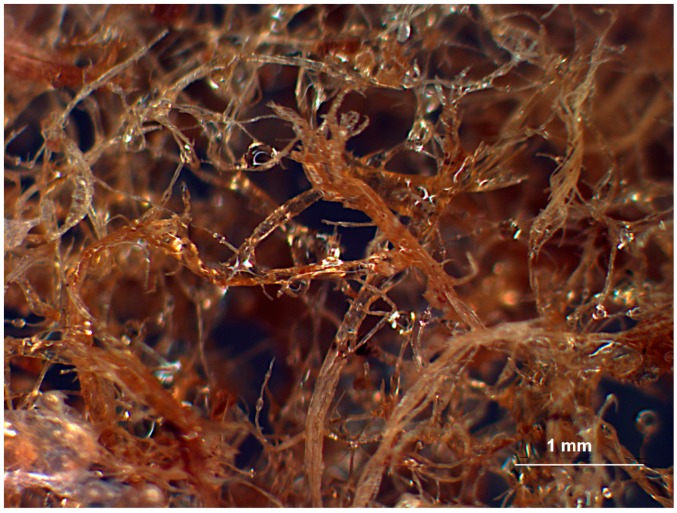
Optical Image of 33% Vinyester resin and MDF fiber.

Solidified vinyl ester-MDF mixtures were imaged in an Omax stereo microscope with magnification of ×10. Figure 8 and Figure 9 show typical optical micrographs of vinyl ester resin/fibre mixtures with 10% and 33% weight fraction of fibers respectively. The glossy surface of fibers suggests a complete coverage of fibres by resin. Since no consolidation pressure has been applied, the mixing and dispersion of fibers was the result of jet impingement mixing of the individual fibers and resin droplets. It is clear from Figure 8 and Figure 9 that the pneumatic conveyor design provides good dispersion of fibers, exposing individual fibers to the stream of sprayed resin. 

A second set of trials of the integrated unit was performed using a two-part polyurethane spray kit: Handi-Foam^®^ E84 (Fomo Products, Inc., Norton, MA, USA). This kit, intended for consumer and professional spray foaming in residential construction, consists of two pressurized tanks (1723.7 kPa) containing isocyanate and polyol components. A spray gun with 5.08 cm (2′′) static mixer was used to mix and spray the PU in a jet towards the test panel. At the same time, the air/fibre stream from the dispersion unit was directed into the resin jet, as depicted in Figure 7. The PU had a rapid cure, resulting in tack free composite foams in less than thirty seconds, with a full cure in 60 min. The polyurethane foams were manufactured with 9%–10% weight fraction of fibres. 

X-ray microtomography of the opaque foam/fibre mixtures were performed using a SkyScan 1172 with a 10 Megapixel camera at a beam voltage of 45 Kilovolts (Bruker microCT, Kontich, Belguim). The size of the samples analyzed was approximately 15 mm × 4 mm × 2 mm (height × length × width). The image resolution was approximately 10 microns. 

**Figure 10:**
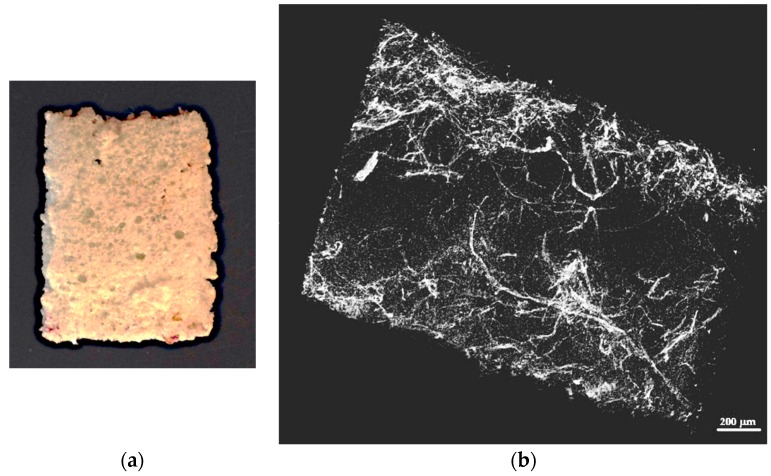
(**a**) Optical; and (**b**) X-ray tomography image of the polyurethane foam with 9% weight fraction of MDF fibers.

Figure 10 is a typical X-ray microtomograph of the resultant polyurethane foam, and has 9% weight fraction of MDF fibers. The fibres show up as light regions in the image, and relatively good fiber dispersion in the foam is apparent in the picture. In this image, which is equivalent to a simple two dimensional X-ray of the sample, the areas devoid of fibres are most likely large bubbles. The fact that the fibres and resin are well mixed is quite remarkable, given that mixing occurred strictly during jet impingement and perhaps also upon impact with the mold surface. 

## 5. Conclusions

A novel dispersion and mixing system useful for working with loose bulk fibres has been designed and preliminary tests of the prototype show promise. The system is designed to disperse short discontinuous fibers in composite spray lay-up process. A pneumatic conveying unit was used to deliver well-dispersed fibres to a jet impingement mixing zone. The compressed air used to drive the pneumatic conveying unit creates the vacuum and turbulence needed to entrain, convey, and disperse clumps of fibres from a hopper full of bulk material. Trials with a 100% pneumatic system indicated that it may be possible to meter a controlled fiber feed rate using air pressure control alone, but in experiments used to produce the first samples, a simple set of interlocking mechanical rollers was used to ensure a consistent flow, and this system worked well. 

Model composite samples containing MDF fibers mixed with vinyl ester resin and polyurethane foam were manufactured. Tomographs and optical images showed a reasonably uniform dispersion of the fibers in both types of specimens, suggesting that jet impingement mixing in free space can be used to generate natural fibre/thermoset composites. Manufacturing composite samples with controlled levels of reinforcing fibre requires equipment capable of simultaneous, coordinated and controlled discharge of fibers and resin. Although this standard has not yet been met, the primary lab experimental set up confirmed that pneumatic dispersion of natural fibers is a promising process for large-scale production of natural fiber thermosetting composites.
